# Seasonal rainfall at long-term migratory staging sites is associated with altered carry-over effects in a Palearctic-African migratory bird

**DOI:** 10.1186/s12898-016-0096-6

**Published:** 2016-10-04

**Authors:** Marjorie C. Sorensen, Graham D. Fairhurst, Susanne Jenni-Eiermann, Jason Newton, Elizabeth Yohannes, Claire N. Spottiswoode

**Affiliations:** 1 Department of Zoology, University of Cambridge, Cambridge, CB2 3EJ UK; 2 Department of Biology, University of Saskatchewan, Saskatoon, S7N 5E2 Canada; 3 Swiss Ornithological Institute, Sempach, Switzerland; 4 NERC Life Sciences Mass Spectrometry Facility, Scottish Universities Environmental Research Centre, Rankine Avenue, East Kilbride, G75 0QF UK; 5 Limnological Institute, University of Konstanz, Mainaustrasse 252, 78464 Constance, Germany; 6 DST-NRF Centre of Excellence at the FitzPatrick Institute, University of Cape Town, Cape Town, South Africa

**Keywords:** Great reed warblers, Long-term staging, Plasma metabolites, Migration, Stable isotopes

## Abstract

**Background:**

An understanding of year-round habitat use is essential for determining how carry-over effects shape population dynamics in long-distance migratory songbirds. The recent discovery of long-term migratory staging sites in many species, prior to arrival at final wintering sites, adds complexity to efforts to decipher non-breeding habitat use and connections between sites. We investigated whether habitat conditions during migratory staging carry over to influence great reed warbler (*Acrocephalus arundinaceus*) body condition at final wintering sites in Zambia. We asked whether the presence/absence and strength of such carry-over effects were modified by contrasting rainfall conditions during 2 years.

**Results:**

First, we found that individuals staging in a dry year had higher corticosterone (CORT_f_) and stable nitrogen isotope values (suggesting higher aridity) than birds staging in a wet year, indicating that regional weather affected staging conditions. Second, we found that carry-over effects from staging habitat conditions (measured via carbon and nitrogen isotopes) to final winter site body condition (measured via scaled mass index and β-hydroxybutyrate) were only present in a dry year, suggesting that environmental factors have consequences for the strength of carry-over effects. Our results also suggest that wet conditions at final winter sites may buffer the effects of poor staging conditions, at least in the short term, since individuals that staged in a dry year had higher scaled mass indices in Zambia than individuals that staged in a wet year.

**Conclusions:**

This study provides a first insight into the connections between long-term migratory staging sites and final wintering sites, and suggests that local environmental factors can modify the strength of carry-over effects for long-distance migratory birds.

**Electronic supplementary material:**

The online version of this article (doi:10.1186/s12898-016-0096-6) contains supplementary material, which is available to authorized users.

## Background

The population dynamics of migratory animals may be affected by factors operating at multiple discrete sites throughout the annual cycle. Long-distance migrants must contend with extreme variation in weather conditions, habitat types and habitat quality, imposed by sites thousands of kilometers apart. This variation can be exacerbated by anthropogenic impacts that disrupt links between specific habitats and crucial periods of the annual cycle [1]. Breeding conditions are well known to have important consequences for population dynamics, but evidence is growing that conditions experienced during non-breeding seasons may be at least as important [2], such that conditions during migratory staging may carry over to affect future breeding success [3]. However, identifying such carry-over effects (defined as processes in one season influencing the success of an individual in the following season) between migratory staging sites has been challenging owing to difficulties in tracking small-bodied migrants across multiple sites throughout the annual cycle [3].

There is strong evidence that winter weather conditions, specifically rainfall effects on food availability, play an important role in phenology and reproductive success of migratory birds, such that low precipitation during the winter has long-term effects on population dynamics [4–7]. However, understanding individual carry-over effects from wintering ground conditions requires knowledge of movements within the non-breeding season and the specific habitats thus encountered. Recently, new light has been shed on the largely unknown winter migratory patterns of long-distance migratory songbirds, using miniature light-level geolocators [8]. Surprisingly, geolocator data show that rather than single wintering sites or “itinerant” winter movements [9], the use of long-term staging sites en route to core wintering grounds may be widespread among migratory songbirds [10–14]. Long-term staging sites complicate our understanding of year-round habitat use for migratory birds because variation in habitat quality among staging sites may have important consequences for population dynamics via carry-over effects on individual survival and condition.

Studying how conditions at multiple sites cumulatively affect year-round individual success is particularly important for Palearctic-African species conservation, management, and effective reserve design, as sub-Saharan wintering migrants are in a state of severe population decline [15, 16]. To begin addressing these shortcomings we studied the great reed warbler (*Acrocephalus arundinaceus*), a 30 g long-distance Palearctic-African migratory songbird that winters widely across sub-Saharan Africa, in southern Zambia. During autumn migration, great reed warblers pause for 2–3 months to moult their flight feathers before continuing on to their final winter sites further south where moult is completed [17]. We focused on rainfall as a potential driver of carry-over effects because its effect on food availability makes it a critical environmental factor for insectivorous birds [18]. We studied great reed warblers during 2 years with contrasting rainfall conditions at staging areas (estimated from isotopic data; see “Results”). Carry-over effects are unlikely to be uniform across years and our understanding of the processes that are capable of modifying their strength remains limited. Natural variation between our study years allowed us to investigate whether the presence and strength of carry-over effects vary depending on environmental conditions.

**Fig. 1:**
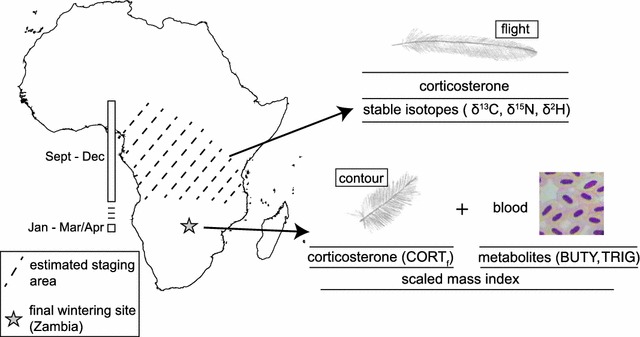
Feather collection, blood sampling, and associated measures of condition for great reed warblers captured in Zambia. We tested whether the presence and strength of carry-over effects varied depending on habitat conditions. *δ* ^15^N, *δ* ^13^C, and CORT_f_ were analysed in flight feathers moulted during staging to assess staging habitat quality, and *δ* ^2^H was added for use in a triple-isotope cluster analysis to estimate staging locations. Body condition at the Zambian winter site was assessed using plasma metabolites in blood, scaled mass index, and CORT_f_ from contour  feathers grown at this site

We first established that differences in rainfall between years affected the conditions birds experienced while staging. To do so, we utilised the fact that great reed warblers replace their flight feathers during staging [19], such that individuals arriving in Zambia carry an isotopic and hormonal signature of the conditions they experienced previously during staging. Specifically, we analysed stable isotope ratios of nitrogen (*δ* ^15^N) and carbon (*δ* ^13^C), and corticosterone (CORT_f_) levels in flight feathers grown on staging grounds (Fig. 1). Both *δ* ^15^N and *δ* ^13^C isotope ratios have been used previously to assess habitat conditions experienced by migratory birds [20–22], trophic position in food webs, or marine versus freshwater ecosystems [23]. *δ* ^15^N increases with greater aridity, especially in Africa [24–26], and *δ* ^13^C decreases as C_3_-dominated plant communities, which are associated with moister and cooler environments, become less common [27]. Corticosterone is a hormone involved in energy management and high levels can indicate compromised individual condition, reflecting a response to stressors such as reduced food availability and poor habitat quality [28–30]. CORT_f_ is an integrated measure of the hormone and reflects conditions experienced over the feather growth period ([31, 32]; for a review, see [33]). We expected that if birds indeed experienced drier conditions during staging in 2011, this would be recorded in their flight feathers as higher *δ* ^15^N values (reflecting higher aridity) and higher CORT_f_ (reflecting a physiological response to poor conditions); however, we expected no difference in *δ* ^13^C values since carbon isotopes are relatively stable between years [34].

**Fig. 2:**
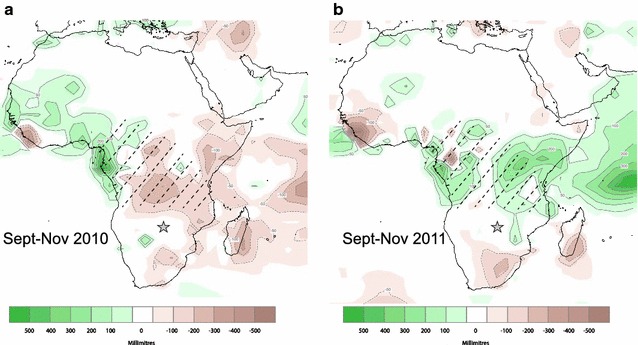
Rainfall conditions experienced by great reed warblers during staging in **a** 2010 (2011 wintering season in Zambia) and **b** 2011 (2012 wintering season in Zambia). *Hashed lines* indicate estimated staging areas from triple-isotope cluster analysis (see “Results” section). Colour range (*brown to green*) indicates the range of rainfall anomalies (below average = *brown*, above average = *green*; NOAA data [42]. The Zambian study site is indicated by a *star*

Second, we asked whether variation in environmental conditions experienced during staging affected the presence and strength of carry-over effects. We used several physiological metrics to estimate condition on wintering grounds. Plasma metabolites are indicators of energetic state: high circulating levels of triglycerides in plasma (hereafter ‘TRIG’) indicate fat deposition and foraging rates over short time-scales [35, 36], whereas high circulating levels of β-hydroxybutyrate (hereafter ‘BUTY’) in plasma indicate dietary fasting, as BUTY is synthesized from fatty acids and largely replaces glucose in fueling metabolism during fasting [37, 38]. We also used scaled mass index [39] to estimate the amount of stored fat. Finally, to gain insight into stress levels on the final wintering site, we measured CORT_f_ levels in body feathers which, unlike flight feathers, are replaced in Zambia after staging (Fig. 1). Since poorer staging conditions should exacerbate differences in quality between individuals, we predicted that in 2011, a year with below-average rainfall, carry-over effects would be stronger than in 2012, a year with above-average rainfall (Fig. 2).

## Results

### Estimated location of great reed warbler staging sites

We captured 26 great reed warblers in 2011 and 38 in 2012. The triple isotope cluster analysis assigned the majority of great reed warbler staging locations to cluster 2 (79 %), corresponding to Angola, Zambia, Zimbabwe, and southern portions of the Democratic Republic of the Congo. Some birds were assigned to cluster 1 (8 %) and cluster 3 (13 %), but none were assigned to cluster 4 which is associated with the Horn of Africa and the north-eastern Sahel region (Table 1; see [40] for a cluster map of Africa). A recent geolocator study of Swedish breeding great reed warblers staging in West Africa found no further migration to southern Africa after staging and moult [17], which concurs with ringing data [41] and suggests that birds wintering in southern Africa do not stage in West Africa prior to reaching their final wintering grounds, and originate from different European breeding grounds. Lemke et al. [17] also found that the mean distance moved after staging was 678 km. Taken together, these results suggest that the most common staging sites for our study population are within 678–2000 km north of the Zambian study site (extent of most of cluster 2 excluding West African component of the cluster), and do not incorporate cluster 4 (East Africa) or West Africa.

**Table 1:** Estimated staging areas for great reed warblers caught in Zambia in 2011 and 2012

Cluster	2011	2012	Years combined
1	3 (12 %)	2 (5 %)	5 (8 %)
2	18 (72 %)	32 (84 %)	50 (79 %)
3	4 (16 %)	4 (11 %)	8 (13 %)
4	0 (0 %)	0 (0 %)	0 (0 %)

Cluster 1: Congo Basin, north Africa, and pockets of west Africa, southern Africa, and Madagascar; cluster 2: Angola, Zambia, Zimbabwe, and southern portions of the Democratic Republic of the Congo but also to extreme west Africa; cluster 3: a broad arc spanning the Atlantic coast of Senegal, south and east toward the Congo Basin, along Africa’s eastern shoreline and westward into Botswana and Namibia; cluster 4: horn of Africa and the north-eastern Sahel region; see Fig. 3 in [40] for details. Staging clusters were estimated via a triple isotope cluster analysis, using isotope values (*δ* ^15^N, *δ* ^13^C, *δ* ^2^H) from tail feathers moulted during staging. The number of birds assigned to each cluster is included along with the corresponding proportion

### Staging site rainfall in 2011 and 2012

Rainfall within estimated staging sites during autumn migration differed between the 2 years of this study. Birds wintering in Zambia in 2011 experienced lower than average rainfall during autumn migration staging, whereas birds wintering in 2012 experienced higher than average rainfall (Fig. 2).

### Does staging rainfall affect conditions experienced by great reed warblers?

**Fig. 3:**
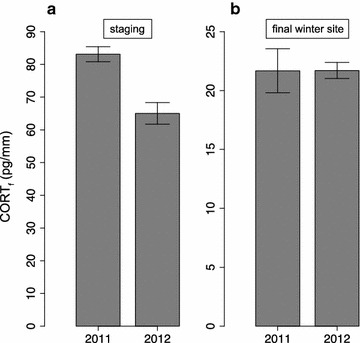
Difference in the hormonal signatures of feathers (CORT_f_) grown on **a** staging (t test on ranked data: t*’*_52.9_ = 6.9, p < *0.0001*; 2011: n = 21, 2012: n = 34) and **b** final wintering grounds (t test on ranked data: t*’*_48.5_ = −0.9, p = 0.35; 2011: n = 24, 2012: n = 36) of great reed warblers in a year with below-average rainfall (2011), and a year with above-average rainfall (2012). *Whiskers* indicate ranges

In support of the NOAA rainfall data (reported in Fig. 2; [42]), isotopic results also suggest that birds indeed experienced drier conditions in 2011 than in 2012: *δ* ^15^N values during staging were higher in 2011, suggesting that more arid conditions were experienced by birds in 2011 than in 2012 (t test: t′_54.4_ = 2.9, p = 0.006; 2011: mean = 9.9 ‰, SE ± 0.26; 2012: mean = 8.9 ‰, SE ± 0.22). In addition, as predicted, staging CORT_f_ (feather corticosterone) was higher for 2011 than for 2012 (t test on ranked data: t′_52.9_ = 6.9, p < 0.0001; 2011: mean = 83.1 pg/mm, SE ± 2.3; 2012: mean = 65.0 pg/mm, SE ± 3.3; Fig. 3) while CORT_f_ from feathers grown on the final wintering grounds in Zambia was similar between years (t test on ranked data: t′_48.5_ = −0.9, p = 0.35; 2011: mean = 21.7 pg/mm, SE ± 1.86; 2012: mean = 21.7, SE ± 0.68; Fig. 3). As expected there was also no difference between years in *δ* ^13^C values from feathers grown during staging (*δ* ^13^C = t test: t′_47.5_ = −0.3, p = 0.75; 2011: mean = −15.7 ‰, SE ± 0.76; 2012: mean = −15.4 ‰, SE ± 0.53).

### Does yearly variation in rainfall affect the strength of carry-over effects?

**Fig. 4:**
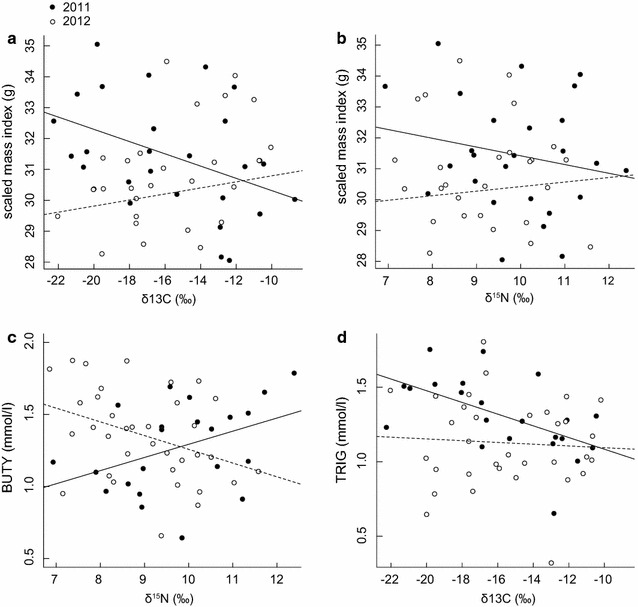
Exploratory analysis for interaction terms that were included in the top model set (top 2 AIC_c_ scores; Table 2); the response variable and parameter of interest were regressed separately for each year. **a** scaled mass index: δ^13^C * year (2011: r^2^ = 0.17, p = 0.03; 2012: r^2^ = 0.03, p = 0.37), **b** scaled mass index: δ^15^N * year (2011: r^2^ = 0.04, p = 0.32; 2012: r^2^ = 0.009, p = 0.6); **b** BUTY: δ^15^N * year (2011: t = 1.83, p = 0.08; 2012: t = −1.34, p = 0.19); **c** TRIG: δ^13^C * year (2011: t = −1.7, p = 0.1; 2012: t = 0.34, p = 0.74). Time at capture and scaled mass index were included as covariates in **c** BUTY and **d** TRIG models (see “Methods” section), *plots* show the raw data

With scaled mass index (on the Zambian final winter site) as the response variable, interaction terms included in the top model set were *δ* ^13^C * year, and, *δ* ^15^N *year. Other models within 2 AIC_c_ scores included staging CORT_f_, with combinations of year or *δ* ^15^N (Additional file 1: Table S1). The interaction term between *δ* ^13^C and year was significant in the multivariate model selected using AIC_c_, whereas the interaction between *δ* ^15^N and year was not (Table 2). To better understand the relationship between *δ* ^13^C and year, we regressed scaled mass index against *δ* ^13^C separately for 2011 and 2012. The relationship between scaled mass index and *δ* ^13^C was negative in 2011 but not in 2012 (2011: r^2^ = 0.17, p = 0.03; 2012: r^2^ = 0.03, p = 0.37; Fig. 4a), indicating that in 2011 birds staging in areas with fewer C_3_ plants (associated with moister, cooler habitats) had lower scaled mass indices in Zambia. *δ* ^15^N was not related to scaled mass index in either 2011 (r^2^ = 0.04, p = 0.32) or 2012 (r^2^ = 0.009, p = 0.60; Fig. 4b).

**Table 2:** Summary of models (in the top 2 AIC_c_ scores) with an interaction term included (see “Methods” section)

Model parameters	Model statistics			Interaction term statistics	
	F	r^2^	p	t	p
Scaled mass index					
Year, carbon, staging CORT_f_, carbon*year	2.75	0.19	*0.04*	2.19	*0.03*
Year, nitrogen, staging CORT_f_, nitrogen*year	2.29	0.17	0.07	1.62	0.11
BUTY					
Year, nitrogen, scaled mass index, time at capture, nitrogen*year	2.83	0.22	*0.03*	2.89	*0.005*
Year, nitrogen, carbon, scaled mass index, time at capture, nitrogen*year	2.35	0.23	*0.05*	2.83	*0.007*
TRIG					
Year, carbon, scaled mass index, time at capture, carbon*year	2.98	0.24	*0.02*	1.48	0.15

Parameters were chosen using AIC_c_ for each response variable separately (scaled mass index, *B*-hydroxybutyrate *BUTY*, triglyceride *TRIG*, final winter site feather corticosterone *CORT*_*f*_. Scaled mass index and time at capture were added as additional covariates for BUTY and TRIG models (see “Methods” section). All covariates were standardized prior to analyses such that effect sizes were comparable

With BUTY as the response variable, every model in the top model set included the interaction term *δ* ^15^N * year (Additional file 1: Table S1). The interaction between *δ* ^15^N and year was significant in both of the selected multivariate models (Table 2). When years were analysed separately and scaled mass index and time at capture were included as covariates (see “Methods” section), the relationship between *δ* ^15^N and BUTY was weakly positive in 2011 but not in 2012 (2011: t = 1.83, p = 0.08; 2012: t = −1.34, p = 0.2; Fig. 4c). This may indicate that in 2011, individuals staging in habitats with higher *δ* ^15^N values (corresponding to higher aridity) had higher BUTY levels (corresponding to increased fat catabolism and fasting) in Zambia.

With TRIG as the response variable, the interaction between *δ* ^13^C * year was included in the top model set. Other models within 2 AIC_c_ scores included year, with combinations of *δ* ^13^C, *δ* ^15^N or staging CORT_f_ (Additional file 1: Table S1). The interaction between *δ* ^13^C and year was not significant in the multivariate general linear model included in the top model set (Table 2). When years were analysed separately with scaled mass index and time at capture included as covariates (see “Methods” section), *δ* ^13^C was negatively related to TRIG in 2011 but not in 2012; however, these relationships were not significant (2011: t = −1.7, p = 0.1; 2012: t = 0.34, p = 0.74; Fig. 4d).

With winter CORT_f_ as the response variable, no interaction terms were included in the top model set (Table 2). Models within 2 AIC_c_ scores included combinations of carbon, nitrogen, and staging CORT_f_ (Additional file 1: Table S1).

The relationships between staging conditions and final winter site conditions in Zambia did not differ between males and females in either 2011 or 2012 (Additional file 1: Table S3).

### Zambian final winter site rainfall and body condition

We tested whether body condition at the final wintering site was better predicted by environmental conditions experienced during staging, or experienced at the final wintering site. Local rainfall conditions at our study site in Zambia were higher in 2011 (December–February total = 736 mm) than in 2012 (595 mm), which was the reverse of rainfall conditions on the staging grounds (Fig. 2). We found that winter CORT_f_, BUTY, and TRIG did not differ between years (winter CORT_f_: t test on ranked data t′_48.5_ = −0.92, p = 0.36; BUTY: ANCOVA F_1,51_ = 0.08, p = 0.79; TRIG: ANCOVA F_1,48_ = 1.06, p = 0.31); however, the great reed warbler population in Zambia in 2012 had a lower scaled mass index (an estimate of stored fat) than the population in 2011 (t test t′_56.3_ = 2.4, p = 0.02). This may suggest that in these 2 years body condition was more strongly related to the most recently experienced rainfall conditions (i.e. at final wintering grounds) rather than those experienced during staging. See Additional file 2 for all supporting data.

## Discussion

Recent research has found that migratory songbirds wintering in sub-Saharan Africa frequently use long-term staging sites before reaching their final destination winter sites. Our results support the hypothesis that local conditions during staging carry over to influence individual condition on the final wintering grounds, but only in years when rainfall conditions are poor. We found that in a drier-than-average year on staging sites (2011), birds had higher CORT_f_ levels and *δ* ^15^N values, respectively reflecting exposure to stronger (or more pervasive) stressors and higher aridity, than birds staging in a year with above-average rainfall (2012; Fig. 3). This indicated that rainfall during staging did indeed affect the local conditions experienced by the population. We then found that individual variation in staging conditions experienced by birds (estimated by *δ* ^13^C and *δ* ^15^N) in 2011 carried over to affect their winter condition in Zambia, as estimated by scaled mass index and BUTY, both of which are likely to influence their overwinter survival and subsequent success on northwards migration. However, in a wetter-than-average year (2012), such individual variation in staging conditions was unrelated to over-winter condition in Zambia, suggesting that when conditions were good, all individuals had access to high quality habitats. Our results demonstrate that differences in local long-term staging conditions may be important for species demography [1]. Moreover, our findings emphasise the challenges in conserving multi-site migration routes and understanding how annual variation in local conditions determine the strength of carry-over effects.

The data support the predictions of our hypothesis; however, they are based on a comparison between 2 years and additional years of study are needed to confirm the generality of the pattern they suggest. It is possible that other differences between years, in addition to rainfall, may have contributed to our observed results. However, rainfall is the most plausible driver given that its connection to food supply is known to be an important environmental driver for the success of wintering migratory birds [18, 43].

### Can good conditions on final wintering sites buffer the effects of poor staging conditions?

Despite having experienced poorer habitat conditions during staging, birds wintering in Zambia in 2011 had higher scaled mass indices than in 2012. One explanation may be that local rainfall conditions at the winter study site were wetter (+141 mm) in 2011 than 2012, and that this difference was enough to counteract drier-than-average staging conditions. Alternatively, poor staging conditions in 2011 might have resulted in differential survival [43]. If only the highest quality birds were able to complete the second stage of migration to final winter sites, this would cause scaled mass index to be higher across the winter population despite the presence of carry-over effects from poor staging conditions. Either way, if rainfall underlies these patterns then good rainfall conditions in Zambia appear to be sufficient to counteract drier-than-average staging conditions, at least in the short term [6]. Whether and how carry-over effects from staging during dry years might affect long-term indicators of success (such as spring migration departure dates and success, survival, and fitness) is an important question for determining the consequences of staging site conditions, and should be a focus of future research exploiting continual improvements in tracking technology.

### Migratory staging and connectivity

Determining the location of long-term staging sites is essential for understanding how staging habitat conditions may contribute to population dynamics. In this study, we used a triple isotope (*δ* ^15^N, *δ* ^13^C, *δ* ^2^H) cluster analysis to assign birds to staging locations. While this method can assign migrants to large clusters in Africa, it lacks the specificity that can be obtained from geolocator studies. Future work that combines geolocator and isotope data will help to improve the applicability of isotopic assignment in Africa. For species with strong migratory connectivity, determining specific staging sites may be essential for determining population responses to conditions during staging; for example, extreme local staging conditions may have specific and dramatic consequences later in the annual cycle. By contrast, for species with weak migratory connectivity, those effects will be diffuse across the species’ range [44]. Great reed warblers seem likely to experience diffuse effects, since breeding and wintering populations appear to be loosely connected [17]. The discovery of multiple long-term staging sites in many species makes studies of migratory connectivity more difficult, since the degree of connectivity with breeding areas could change over the non-breeding season as birds shift locations. Determining how staging locations are connected to both breeding and wintering grounds is important for predicting the consequences of poor rainfall or drought conditions.

## Conclusions

This study supports the hypothesis that habitat conditions on distant long-term staging sites can influence individual condition at final wintering sites, and suggests that these effects are likely to be strongest when conditions are relatively poor. Longitudinal studies are needed to confirm whether the latter pattern is general. Our results suggest that when conditions on final wintering sites are good, individuals may be able to buffer the effects of poor staging conditions in the short term; however, differential survival due to poor staging conditions may also account for this pattern. Research determining variation in the strength of carry-over effects between years has been limited (but see [18, 45, 46]), and to our knowledge this study is the first to assess carry-over effects between recently-discovered long-term staging sites and final wintering sites. Long-term staging sites add complexity to our understanding of population dynamics for declining migratory species especially in the context of global change, in which habitat loss (e.g. owing to agriculture) and unpredictable events outside the reproductive period such as food shortages (e.g. owing to drought) are likely to increase. Our results provide further incentive for logistically challenging endeavours that aim to quantify year-round habitat use and disentangle interactions between multiple environmental factors and sites.

## Methods

### Study site and field data

Fieldwork was conducted in southern Zambia on and around Muckleneuk Farm, near Choma, in ca. 280 ha of mesic grass and reed-bed habitat around each of two watercourses and associated dams (site 1: 16°39′S, 27°00′E; site 2: 16°37′S, 26°59′E), from January to April 2011 and 2012. The surrounding habitat is thornbush savannah and broad-leaved woodland with areas of tobacco, maize, and wheat cultivation. Great reed warblers are widely distributed across their European breeding grounds and sub-Saharan wintering grounds, and absent only from the southwestern half of South Africa and parts of Botswana and Namibia [47].

Great reed warblers were captured using 8–10 mist nets erected daily from 0530 to 1100 h. Birds were captured without the use of playbacks in order to avoid biases towards the most aggressive individuals. At the time of capture, all individuals were weighed with an electronic scale (± 0.01 g), and tarsus length, bill length, and wing length were measured with digital calipers (± 0.1 mm). To estimate body condition, we calculated the scaled mass index from our morphometric data. This approach scales the mass of all individuals to that expected if they were all of identical body size, and is preferable to the more traditionally used residuals of mass on length [39]. With this approach, a single measure of size is recommended rather than a principal component analysis (PCA) of multiple measures [39], so we used wing length as our measure of structural size since it was correlated most strongly with weight (wing: r = 0.46, p < 0.001; bill: r = 0.35, p = 0.004; tarsus: r = −0.03, p = 0.85, n = 61). Within 10 min of capture, a 100 μl blood sample was taken for molecular sexing and metabolite analyses (see “Plasma metabolites” section below); thereafter, 5–8 contour feathers (collected from the upper breast) and the 5th right rectrix feather were sampled for isotope and CORT analyses (see below). Because great reed warblers are sexually monomorphic, sex was determined using a molecular technique. Primers for molecular sexing were modified from the primers 2550F and 2718R first described by [48]; see [49] for details. Juveniles (first-winter birds) may initiate staging moult on average 15 days later than adults (51 day total moult duration; [19]), which could cause differences in isotope signatures of feathers; however, this effect is likely to be minimal given the total length of moult. Juveniles were separated from adults by using tongue spot distinctiveness, eye, and tarsus colouration (for details see [50]). Indeed, we found no difference in isotope signatures between adults and juveniles in our data (*δ* ^15^N: t test t′_24.1_ = 0.69, p = 0.5; *δ* ^13^C: t test t′_23.8_ = 0.13, p = 0.9). Therefore, all individuals (2011 = 7 juveniles, 19 adults; 2012 = 9 juveniles, 28 adults) were pooled for analysis.

### Rainfall measures

Figure 2 shows precipitation during migratory staging (September–November averages) in the northern-hemisphere autumn of 2010 (hereafter ‘2011’ as this is when sampling took place in Zambia) and 2011 (hereafter ‘2012’). To quantify differences between years, we used 3 month precipitation anomalies from the NOAA Climate Prediction Center’s CAMS_OPI dataset (http://www.ncdc.noaa.gov/cag/mapping/global; Accessed May 15 2014; [42]), which merges observations from rain gauges with precipitation estimates from a satellite algorithm. Anomalies are expressed as the deviation from the 1979–2000 mean value. September–November averages are presented in order to include rainfall just prior to autumn arrival on staging sites (end of September to early October; [17], since rainfall during this time is likely to be important given the time lag observed between precipitation and primary productivity [51]. We used farm records (Muckleneuk Farm, 16°38′S, 27°00′E) to assess local rainfall at the final winter site in Zambia; rainfall was recorded daily in mm using a rain gauge (from December to February 2011 and 2012).

### Isotope analysis

#### Carbon and nitrogen

Prior to all isotope analyses, feathers were washed in 2:1 chloroform:methanol solution for 24 h then rinsed with distilled water and left to air dry for 24 h. Sub-samples of 0.8–0.9 mg were weighed into tin capsules. Samples were combusted in a Pyrocube (Elementar, Hanau) elemental analyser. The resulting CO_2_ and N_2_ were separated by gas chromatography and admitted into the inlet of a Micromass (Manchester, UK) Isoprime isotope ratio mass spectrometer (IRMS) for determination of ^13^C⁄ ^12^C and ^15^N⁄ ^14^N ratios. Measurements are reported in *δ*-notation (*δ* ^13^C and *δ* ^15^N, respectively) relative to the Pee Dee Belemnite (PDB) for carbon and atmospheric N_2_ for nitrogen in parts per thousand deviations (‰) using the formula *δ* (‰) = 1000 × [R_sample_/R_standard_−1]. Two sulfanilamides (Isoprime internal standards) and two Casein standards (in house standard) were used for every seven unknowns in sequence. Internal laboratory standards indicated measurement errors (SD) of ±0.05 ‰ for *δ* ^13^C, 0.12 ‰ for *δ* ^15^N.

#### Hydrogen

Cleaned samples of approximately 0.2 mg were weighed into silver capsules and pyrolysed in an Elementar (Hanau, Germany) Pyrocube elemental analyser over glassy carbon. The resulting H_2_ was admitted into the source inlet of a Thermo (Bremen, Germany) XP Plus mass spectrometer. Measurements are reported in *δ*-notation relative to the international standard SMOW (Standard Mean Ocean Water). Organic materials involving H not bonded to carbon will readily exchange H with water vapour [52]. Subtraction of this exchangeable hydrogen is attained using standards with known non-exchangeable (i.e. indigenous) hydrogen isotope compositions. These are CFS (chicken feathers), BWB-II (bowhead whale baleen), and ISB (black kittiwake feathers; for CFS and BWB-II see [53]; for ISB see [54]. Replicate measurements of these standards imply measurement errors (SD) of around 2 ‰ for δ^2^H.

### Triple-isotope cluster analysis

We used the isotope signature of great reed warbler tail feathers, grown during staging, to determine the most likely staging locations in Africa. Specifically, we used a three-isotope (*δ* ^2^H, *δ* ^13^C, *δ* ^15^N) cluster analysis developed by [40] to assign birds to one of four clusters in Africa. This method uses isoscapes (i.e. maps of isotopic variation produced by iteratively applying models across regions of space) to estimate the origin of moulted feathers [55]. The clusters were developed by examining previously published plant *δ* ^13^C and *δ* ^15^N [56, 57] isoscapes and the long-term hydrologic *δ* ^2^H [55] isoscapes of Africa, to obtain information on geographic structure in order to find “natural” groupings in multivariate space (Fig. 2; see [40] for details). Linear discriminant function analysis was then used to derive algorithms that predict the posterior probability that a sample with a given multi-isotope composition could have originated from any given cluster within Africa, given the predicted ranges for feather *δ* ^2^H, *δ* ^13^C, and *δ* ^15^N (see [40] for details). Error estimates were derived from [58]. All analyses were conducted within R version 2.15.0 (R Development Core Team 2012) applying algorithms derived from [40] by means of script provided by Steven Van Wilgenburg.

### Plasma metabolites

Blood samples were collected into heparinized capillary tubes from the brachial vein and were centrifuged for 10 min (maximum speed of 8000 rpm) within 4 h of collection. The separated plasma was frozen at −20 °C until analysis. Samples were transported on ice to the Schweizerische Vogelwarte, Sempach, Switzerland, for analyses in May 2012. All metabolites were determined in the plasma using standard test-combinations, the Wako Auto-Kit for 3-hydroxybutyrate (cyclic enzymatic method; BUTY), and the enzymatic colorimetric test for triglycerides (TRIG) including free glycerol (Invicon HIT 917, PAP-method). Dilution curves of the great reed warbler plasma were run for both metabolites to test for linearity. The triglyceride assay was adapted to small volumes (5–10 µl). All samples were measured in duplicates. TRIG and BUTY values were linearly negatively related (r^2^ = 0.06, p = 0.05); this suggests that the inclusion of free glycerol in our test of triglycerides did not obscure true triglyceride values [36].

### Feather corticosterone

Corticosterone (CORT) was extracted from feathers using a methanol-based procedure [59] that has been replicated successfully in passerines [60, 61]. A single tail feather per bird was collected and CORT thus extracted. Because multiple contour feathers were collected from all individuals, two contour feathers per bird were extracted to ensure that CORT_f_ measurements were well within limits of detectablility [62]. Following removal of the calamus, the length of each feather sample (proximal cut to distal tip of vane) was measured flat against the edge of a ruler. Feather samples were then placed in glass vials and cut into tiny pieces (c. 5 mm^2^). Ten mL of methanol (HPLC grade; Fisher Scientific, Fair Lawn, NJ, USA) was added to each vial and samples were sonicated at room temperature for 30 min, followed by overnight incubation at 50 °C. Using a glass funnel fitted with polyester fibre, vacuum filtration was employed to separate the methanol extract from the feather pieces. Collected methanol extracts were evaporated and residues were reconstituted in 600 µL of phosphate-buffered saline (0.05 M, pH 7.6) and frozen at −20 °C until analysed by radioimmunoassay (RIA). Samples were extracted in two batches and the recovery efficiency of each extraction was assessed by spiking three sample extracts with approximately 5000 c.p.m. of ^3^H-labelled CORT. The average recoveries were 98.6 and 93.2 %, and final values were adjusted for recoveries. Serial dilutions of feather extracts were parallel to the CORT standard curve. Samples were analysed in duplicate in two RIAs using a commercial antiserum (Sigma-Aldrich, St. Louis, MO, USA; product #C8784). The variability of each assay was assessed using six replicates of the same internal standard; average intra-assay coefficient of variation (CV) was 7.4 (SD = 1.1) % and inter-assay CV was 5.5 %. Average detection limit (%B/B_0_) was 11.0 (SD = 1.5) pg CORT/100 µl of extract and all samples were above detection limits. CORT_f_ measurements were corrected for total feather sample length, reflecting the time-dependent deposition of CORT into feathers, and were thus expressed as pg CORT/mm [31, 59, 63]. CORT_f_ values are likely determined by the interaction between plasma CORT values and the dynamics of feather growth [33]. Although we cannot be sure that the higher CORT_f_ values during staging in 2011 vs. 2012 were due to decreased feather growth rates, higher plasma CORT levels, or both, responses to poor environmental conditions would likely result in higher CORT_f_ values in all of these scenarios [33], though future work is needed to fully understand the mechanism in our study species.

### Statistical analysis

We analysed each response variable separately (scaled mass index, TRIG, BUTY, final winter site CORT_f_). We used Akaike’s Information Criterion corrected for small sample sizes (AIC_c_; [64]) to select from the following predictors *δ* ^13^C, *δ* ^15^N, staging CORT_f_, and interaction terms between each parameter and year. Because a difference between years was the a priori prediction for our hypothesis, if an interaction term was included in the top model set (within 2 AIC_c_ scores) we constructed multivariate general linear models of all top models including interaction terms (Table 2), and performed exploratory analysis by regressing the response variable and parameter of interest separately for each year (see “Results” section; Fig. 4). For TRIG and BUTY models, time at capture was included as a covariate since this has been shown to be related to plasma metabolite levels [65, 66]; in addition, scaled mass index was included as a covariate in order to assess plasma metabolites as a dynamic measure of change in body condition rather than a static measure of current state, such as scaled mass index [36]. All covariates were standardized prior to analyses such that effect sizes were comparable. All models residuals conformed to normality assumptions for parametric statistics; therefore, response variables were not transformed prior to analysis. Model averaging was not conducted, as sample sizes were not large enough for robust analysis [67].

Three TRIG data points had Cook’s distances substantially higher than the mean (mean = 0.02 ± 0.008 SE; sample 1 = 0.21, sample 2 = 0.18, sample 3 = 0.34; [68]). All three outlying TRIG concentrations were measured within 2 weeks of the start of spring migration, such that these high TRIG concentrations were likely from individuals that had begun pre-migratory fattening and do not represent baseline winter feeding conditions. These three values were therefore removed from the analyses, but conclusions were unchanged if they were left in the dataset.

Sex was not included in the main models due to low residual degrees of freedom. Therefore, to determine whether carry-over effects differed between females and males, separate general linear models were instead run for each year, with an interaction term between sex and all three predictor variables (*δ* ^15^N, *δ* ^13^C, and staging CORT_f_) included.

To test for differences between groups we used unequal variances (Welch’s) t tests; when data did not meet assumptions of normality we ranked the data, following [69]. To test for differences in plasma metabolite values between years we used ANCOVA and included scaled mass index and time at capture as covariates.

## Additional files

10.1186/s12898-016-0096-6 This file contains three tables: the full AIC_c_ ranked candidate model set (2AIC_c_ scores) of the relative importance of staging parameters on final winter site body condition (δ^15^N, δ^13^C, feather corticosterone, and interactions with year); **Table S2:** full results of the top multivariate general linear model with interaction terms included for each response variable (scaled mass index, BUTY, TRIG, and staging CORT_f_); and **Table S3:** full results of models to test for sex differences in carry-over effects between years.

10.1186/s12898-016-0096-6 The raw data collected from great reed warblers at the Zambian study site. Information includes year, sex, age (1 = juvenile, 2 = adult) time at capture (time.count = minutes since 0600 h), winter CORT_f_ (feathercort.C), staging CORT_f_ (feathercort.F), BUTY, TRIG, scaled mass index, δ^13^C, δ^15^N, *δ* ^2^H, and the estimated isotope cluster.

## Abbreviations

CORT: corticosterone
CORT_f_: feather corticosterone
*δ*^15^N: nitrogen isotope
*δ*^13^C: carbon isotope
TRIG: triglyceride
BUTY: β-hydroxybutyrate
