# Supplementary material for: Seasonal rainfall at long-term migratory staging sites is associated with altered carry-over effects in a Palearctic-African migratory bird
Source: BMC Ecol. 2016 Oct 4;16:41. doi: 10.1186/s12898-016-0096-6 (PMC5050568; doi:10.1186/s12898-016-0096-6)
Supplement: Supplementary file 1 — 10.1186/s12898-016-0096-6 This file contains three tables: the full AICc ranked candidate model set (2AICc scores) of the relative importance of staging parameters on final winter site body condition (δ15N, δ13C, feather corticosterone, and interactions with year); Table S2: full results of the top multivariate general linear model with interaction terms included for each response variable (scaled mass index, BUTY, TRIG, and staging CORTf); and Table S3: full results of models to test for sex differences in carry-over effects between years. [file 12898_2016_96_MOESM1_ESM.docx]

**Supplementary material**

**Table A1**: Full AIC_c_ ranked candidate model set (2AIC_c_ scores) showing relative importance of the following parameters on the body condition of great reed warblers at their final wintering site near Choma, Zambia: staging site: δ^15^N, δ^13^C, feather corticosterone, and interactions with year.

**Table A2.** Full results of models in the top model set (top 2 AICc scores) that include an interaction term. Parameters were chosen using AICc for each response variable (scaled mass index, B-hydroxybutyrate (BUTY), triglyceride (TRIG), final winter site feather corticosterone (CORT_f_); see Methods). All covariates were standardized prior to analyses such that effect sizes were comparable.

**Table A3**. Tests for potential sex differences in carry-over effects. General linear models of each response variable (scaled mass index, final winter site CORT_f_, BUTY, TRIG) were separated by year (see Methods), with interaction terms between sex and all three predictor (carbon, nitrogen, staging CORT_f_) variables included. No sex differences were detected. *P* values were adjusted using Bonferroni correction.
